# Eye anomalies and neurological manifestations in patients with *PAX6* mutations

**Published:** 2009-10-22

**Authors:** Yin-Hsuan Chien, Hsiang-Po Huang, Wuh-Liang Hwu, Yin-Hsiu Chien, Tseng-Ching Chang, Ni-Chung Lee

**Affiliations:** 1 Department of Pediatrics, Taipei City Hospital, ZhongXing Branch, Taipei, Taiwan; 2 Department of Medical Research, National Taiwan University Hospital and National Taiwan University College of Medicine, Taipei, Taiwan; 3 Department of Medical Genetics, National Taiwan University Hospital and National Taiwan University College of Medicine, Taipei, Taiwan; 4 Department of Pediatrics, National Taiwan University Hospital and National Taiwan University College of Medicine, Taipei, Taiwan

## Abstract

**Purpose:**

Mutations in the paired box 6 (*PAX6*)**gene cause a wide variety of eye anomalies, including aniridia. *PAX6* mutations are not well described in the Chinese population so this study is aimed at exploring the role of *PAX6* mutations in Taiwanese patients with congenital eye anomalies.

**Methods:**

Seventeen patients with single or multiple congenital eye anomalies were enrolled. Genomic DNA was prepared from venous blood leukocytes, and the coding regions of *PAX6* were analyzed by PCR and direct sequencing. Clinical manifestations of the patients were then correlated to *PAX6* mutations.

**Results:**

Five *PAX6 *mutations were identified in one case each. Three mutations c.317T>A (p.L106X), c.142–1G>T, and c.656del10 (p.Q219QfsX20) were novel and the other two, c.331delG (p.V111SfsX13) and c.949C>T (p.R317X), have been reported. All five cases had aniridia; three had other eye anomalies; and four had developmental delay. Only one case had other affected family members. In the ten cases that had no *PAX6* mutation, only one had aniridia.

**Conclusions:**

Both novel and known *PAX6* mutations were identified in the current study, and *PAX6* mutations were closely associated with aniridia. Absence of a positive family history does not exclude *PAX6* mutation. The frequent occurrence of developmental delay in patients with *PAX6* mutation argues for a prompt diagnosis of the disease.

## Introduction

Aniridia is a rare ocular anomaly characterized by defects of iris tissue, ranging from mild iris hypoplasia to almost total absence of iris [1]. It occurs with an incidence of 1:64,000 to 1:100,000 [2,3]. Two-thirds of the cases show autosomal dominant inheritance with complete penetrance, while others are sporadic [3–5]. Clinical manifestations of patients varied from isolated iris involvement to panocular anomalies involving the cornea (opacity), anterior chamber angle (glaucoma), lens (dislocation and/or cataracts), retina (foveal dysplasia), and macular and optic nerve (hypoplasia) [2]. Aniridia can be associated with Wilms tumor; aniridia, genitourinary disorders, and mental retardation (WAGR syndrome); Gillespie syndrome (aniridia, cerebellar ataxia, and mental deficiency); absent patella; unilateral renal agenesis and mild psychomotor retardation; congenital adrenal hypoplasia and dysmorphism; sensorineural deafness; Marfan syndrome; Smith-Opitz syndrome; Biemond syndrome; XXXXY chromosomal anomalies; or Badet–Biedl syndrome [2,6–13].

Several genes, including the paired box 6 (*PAX6*), paired-like homeodomain 2 (*PITX2/RIEG1*), forkhead box C1 (*FOXC1/FKHL7*), SIX homeobox 3 (*SIX3*), HESX homeobox 1 (*HESX1/RPX)*, paired-like homeodomain 3 (*PITX3)*, cone-rod homeobox* (CRX*), guanylate cyclase 2D, membrane (retina-specific; *GUCY2D*/*RETGC1*), peripherin 2* (PRPH/*RDS), retinal pigment epithelium-specific protein 65kDa (*RPE65*), paired box 2 (*PAX2*), paired box 3 (*PAX3*), microphthalmia-associated transcription factor (*MITF*), jagged 1 (*JAG1*), and retina and anterior neural fold homeobox (*Rx*), and *FOXC1*, are important for eye embryogenesis [1,14,15]. Among them, *PAX6*, *PITX2*, *FOXC1*, and *FLHL7* are associated with iris defects. Haploinsufficiency or dominant negative mutation of *PAX6* leads to aniridia, congenital cataract, Peter’s anomaly, Gillespie syndrome, and midline fusion defects, while complete deficiency of *PAX6* leads to anophthalmia [1,3,4]. Mutations of *PITX2* result in Rieger’s syndrome (anterior segment abnormalities, glaucoma, tooth anomalies, umbilical stump abnormalities); mutations of *FOXC1* result in Rieger’s syndrome (type 3); mutations of *FKHL7* cause anterior segment anomaly with glaucoma (abnormal iridocorneal angle differentiation, iris stromal hypoplasia, and elevated intraocular pressure/glaucoma) [1,14–17].

*PAX6* is a transcriptional regulator in the early development in the ocular system, central nervous system, and gastrointestinal system [5,18]. This gene contains 14 exons and encodes a 422-amino acid polypeptide containing two DNA-binding domains, a bipartite paired domain, and a paired type homeodomain [4]. The paired domain, which is coded by exons 5–7 of *PAX6*, has two subdomains: the relatively conserved 74-amino acid NH_2_-terminal subdomain and the more divergent 54-amino acid COOH-terminal subdomain. The latter subdomain is a common place for mutations [4,19]. Currently there are around 500 mutations that have been reported (Human PAX6 Allelic Variant Database [HPAVD]) [20]. Most *PAX6* nonsense mutations lead to aniridia, while missense mutations are related to foveal hypoplasia, congenital cataracts, or anterior segment anomalies [21,22].

There has been no systemic study for *PAX6* mutations in the Chinese population [23–25]. In this study, we analyzed the coding sequences of *PAX6* in 17 patients with eye anomalies. Three novel and two known heterozygous mutations were detected. Only one patient had other affected family members, but intrafamilial variation was prominent.

## Methods

From 2003 to 2009, 17 patients (nine males and eight females) with single or multiple congenital eye anomalies diagnosed in two hospitals were enrolled in the study after informed consent. They were healthy except for their eye and neurological deficits. The study protocol included slit lamps and neurological examinations, brain Magnetic Resonance Imaging (MRI), pedigree analysis, and *PAX6* gene analysis. Genomic DNA was isolated from 5 milliliters of venous blood using a QIAamp DNA blood mini kit (Qiagen®, Hilden, Germany). *PAX6* coding regions and their flanking intronic sequences were amplified by PCR (Table 1). The PCR products were purified by Gel-M^TM^ Gel Extraction System (Viogene^®^, Taipei, Taiwan) and analyzed by direct sequencing using the ABI Prism Big Dye dideoxy chain terminator cycle sequencing kit and the ABI Prism 310 genetic analyzer (Applied Biosystem, Foster City, CA). *PAX6* cDNA was numbered starting from the translation initiation site (NM_000280.3). Mutations were confirmed by sequencing from the opposite strand and by co-segregation of the lesion and disease within the family. Phenotypes of the patients were retrieved from the medical charts. Clinical manifestations of patients were then correlated to *PAX6* mutations.

**Table 1:** Primers used to amplify the *PAX6 *gene.

**Exon**	**Amplified length (bp)**	**Forward primer (5’ to 3’)**	**Reverse primer (5’ to 3’)**
1	645	GCATGTTGCGGAGTGATTAG	CTCCTGCGTGGAAACTTCTC
2	645	GCATGTTGCGGAGTGATTAG	CTCCTGCGTGGAAACTTCTC
3	676	AGAGAGCCCATGGACGTATG	GTCGCGAGTCCCTGTGTC
4	676	AGAGAGCCCATGGACGTATG	GTCGCGAGTCCCTGTGTC
5	373	TGAGGATGCATTGTGGTTGT	GAAATGAAGAGAGGGCGTTG
6	388	CGTAAGCTTGTCATTGTTTAATGC	AGAGAGGGTGGGAGGAGGTA
7	333	GGTTGTGGGTGAGCTGAGAT	AAGCCCTGAGAGGAAATGGT
8	355	GGCTGTCGGGATATAATGCT	CAAAGGGCCCTGGCTAAAT
9	385	AGGTGGGAACCAGTTTGATG	TGGGACAGGTTAGCACTGTGT
10	537	AGCAGTGGAGGTGCCAAG	TCTCAAGGGTGCAGACACAG
11	537	AGCAGTGGAGGTGCCAAG	TCTCAAGGGTGCAGACACAG
12	345	CAGACTTGTTGGCAGAGTTCC	TAAACACGCCCTCCCATAAG
13	373	TTTCTGAAGGTGCTACTTTTATTTG	CGGCTCTAACAGCCATTTTT

## Results

Of the 17 indexed patients, five had *PAX6* mutations. All five patients had aniridia, and two of them had fovea hypoplasia (Table 2). The five heterozygous mutations are c.142–1G>T, c.317T>A (p.L106X), c.331delG (p.V111SfsX13), c.656del10 (p.Q219QfsX20), and c.949C>T (p.R317X) (Table 2). Mutation c.949C>T and c.331delG have been reported previously [26]. C.949C>T is widespread in different countries, while c.331delG is found only in the USA. Mutation c.317T>A, c.142–1G>T, and c.656del10 have not been previously reported. Both c.317T>A and c.656del10 produce prematurely stopped polypeptides. Mutation c.142–1G>T is located at the splicing consensus sequence of intron 5b and is likely to produce splicing errors similar to reported mutations c.141+1 G>A, c.141+2T>C, and c.142–2A>G [20,27].

**Table 2:** Genotypes, eye anomalies, and extraocular manifestations in patients with a *PAX6* mutation.

**No**	**Inheritance**	Nucleotide change*	Predicted protein change*	**Location**	**Previously reported**	**Eye anomalies**	**Extraocular manifestations**
1	sporadic	c.317T>A	p.L106X	Exon 6	no	aniridia, foveal hypoplasia, pendular nystagmus	
2	Familial	c.949C>T	p.R317X	Exon 11	PAX6 database**	aniridia, cataract, nystagmus	congenital hip dislocation, developmental delay
3	sporadic	c.331delG	p.V111SfsX13	Exon 6	PAX6 database**	aniridia	developmental delay
4	sporadic	c.142–1G>T	Abnormal splicing	Exon 5b	no	aniridia, nystagmus	mild developmental delay
5	sporadic	c.656del10	p.Q219QfsX20	Exon 8	no	aniridia, horizontal pendular nystagmus, foveal hypoplasia	developmental delay

The asterisk indicates that only one mutation is present in each patient with an autosomal dominant disease. The double asterisk refers to the Human PAX6 Allelic Variant Database (HPAVD).

**Figure 1:**
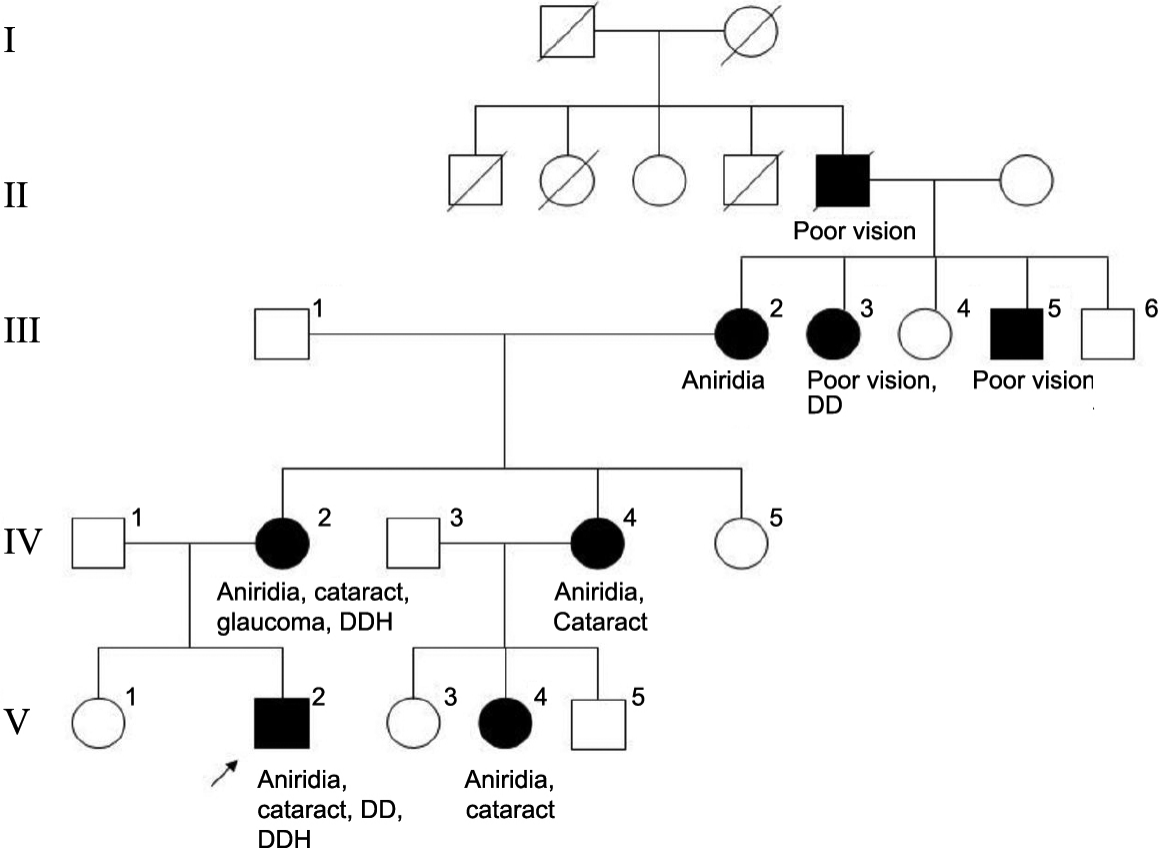
Pedigree of patient 2. The pedigree has been modified for privacy by changing the sequence of the family members. DD, developmental delay; DDH, Developmental dysplasia of hip

Among the five patients, only patient 2 had other affected family members. He and his mother accepted *PAX6* gene analysis and both of them had the c.949C>T mutation (Figure 1. V-2, IV-2). There were eight individuals in this four-generation family who had congenital eye anomalies (Figure 1). Their anomalies included bilateral aniridia, cataract, glaucoma, and jerk horizontal nystagmus (Figure 1). They all had intact retina, choroids, and optic nerve. All other patients, except patient 2, were sporadic. Interestingly, four cases (patient 2 to 5) had delays in gross motor, fine motor, language, and cognition to a variable extent. Patient 2 could not sit until 10 months of age and started babbling only after 1 year of age. After the correction of a congenital hip dislocation and aggressive physical therapy, he walked at 19 months of age and climbed stairs with assistance at the age of 2 years. Pincer grasp was not observed until 12 months of age. Patient 3 walked with support at the age of 13 months and said “papa” and “mama” at 21 months. His brain echo was normal. Patient 4 was noted to have a small subependymal cyst at birth and head lag at age 4 months, but he did not return for follow up thereafter. Patient 5 could not sit up or turn over at the age of 7 months.

Phenotypes of the 12 *PAX6* mutation-negative cases are summarized in Table 3. Only one of the 12 cases without *PAX6* mutation had aniridia. Five of them had microphthalmos, three had dysmorphic facial features, and two had developmental delay.

**Table 3:** Phenotypes of patients who have eye anomalies but no *PAX6* mutation.

**Associated organ**	**Phenotypes**	Number of cases*
Eye	Microphthalmos	5
	Microcornea	2
	Eyelid coloboma	2
	non-specified eye anomaly	2
	Aniridia	1
	Cornea whitish plague, Congenital cataract, Choridal coloboma, Anophthalmos, Strabismus	One each
Central nervous system	Developmental delay	2
	Ventriculomegaly	2
	Periventricular cyst, Hemiparesis, Seizure, Corpus callosum hypoplasia	One each
Genitourinary	Small kidney	2
	Vesiculoureteral reflux, Cryptorchidism	One each
Cardiac	Coarctation of aorta, Patent ductus arteriosus	One each
Skeletal	Congenital hip dislocation	1
Others	Dysmorphism	3
	Choanal atresia, Transient congenital hypothyroidism	One each

The total number of patients = 12

## Discussion

In this study we identified five *PAX6* mutations in 17 patients with congenital eye anomalies, resulting in a mutation detection rate of approximately 30% (5/17) in all patients with congenital eye anomalies or 83% (5/6) in patients with aniridia. Our detection rate is comparable with previous reports that 30%–80% of patients with aniridia have *PAX6* mutations [28–30].

There were eight reports, four in English and four in Chinese, each mentioned a *PAX6* mutation in one Chinese family with aniridia. The reported mutations, including c.1286delC (c.924delC), c.483del9 (c.121_129del9), IVS10+1G>A, c.1080C>T (c.718C>T), and c.857delG (c.495delG) [23,24,31–36], surprisingly none occurred in our population. That each mutation occurs independently demonstrates the great diversity of *PAX6* mutations in the Chinese population. The large proportion of sporadic cases in the current study also suggests the diversity of *PAX6* mutations.

In our cohort, 80% (4/5) of patients with the *PAX6* mutation had developmental delay. This high incidence of developmental delay is unexpected. Patients with aniridia and neurologic problems were more linked to WAGR syndrome (75% have mental retardation), Gillespie’s syndrome, chromosome anomalies, or *PAX6* gene duplication [2,4,37–39]. Although we did not exclude large fragment gene deletions in the current study, none of our patients had syndromic aniridia. Deletions not detectable by DNA sequencing and associated with isolated aniridia have been reported, but they are present only in a small fraction of patients [40,41].

Several *PAX6* mutations have been associated with mild mental retardation (c.-129+2T>A, c.111_2013;141ins, R44X, S74G, I87R, S119R, Q135X, W257X, C719A, c.1267dupT, and 1.3 Mb deletion from 3′ UTR of *PAX6* gene at 11p14.1-p13) [20,29,42–46]. One patient with c.-129+2T>A mutation had hand tremors and learning disabilities [6]; a boy with c.111_141ins had intellectual impairment [29]; microcephaly, developmental delay, and several minor dysmorphic features were noted in the sporadic patient with I87R mutation [20]; one large family with S74G mutation showed neurodevelopmental defects with or without other associated brain anomalies [46]. Occasionally, mental retardation occurred in only a portion of the affected family members [20,42]. In the Human *PAX6* Allelic Variant Database, one of the three cases with S119R mutation had a learning disability and behavioral change; one of the 20 cases with c.1267dupT mutation was recorded to have developmental delay and autistic behavior. The mutations discovered in our series (c.142–1G>T, c.317T>A, c.949C>T, c.331delG, and c.656del110) are different from these reported cases with mental retardation. However the mild developmental delay in this study could have been neglected by other studies because of the vision problems of patients. The global delay in case 2 could not be explained by his vision problem or hip dislocation. Other cases in the current study also involved only gross motor or speech problems, which were difficult to explain by poor visual activity.

*PAX6* gene expression is seen after the end of gastrulation in the anterior neural plate [15]. In a mouse model, *Pax6* is widely expressed in the developing eye (optic cup, lens, and overlying surface ectoderm) and in specific regions of the developing brain (frontal cortex, epithalamus, ventral tagmental area, pons, external granular layer of cerebellum, fovea isthmi, olfactory bulb, septum, olfactory neuroepithelium) [47,48]. *PAX6* has been suggested as being expressed in conjunction with other *PAX* family members in the early regionalization of the brain [47,48]. Recently, the interactions of *Pax6* with developing neocortex transcription factors T-box brain gene 1 (*Tbr1*), eomesodermin homolog (*Tbr2*), neurogenin 2 (*Ngn2*) and achaete-scute complex homologue 1 (*Mash-1*) further demonstrate the role of *PAX6* in the developing neocortex [49–51]. The *PAX6* heterozygous mouse has absent olfactory bulb, decreased cortical neurons and cortical plate thickness, and altered dorsoventral patterning of the forebrain [45]. In patients with *PAX6* mutation, polymicrogyria, absence of pineal gland, and lack of the anterior commisure have all been reported [52,53]. Therefore, it is possible that patients with *PAX6* mutation have neurologic manifestations.

In conclusion, we demonstrated the mutation spectrum and neurologic manifestations of patients with *PAX6* mutation in Chinese. Most patients with aniridia had *PAX6* mutations. Other associated problems, such as developmental delay and even congenital hip dislocations, may also be important. Therefore, detailed neurologic examination and close observation of development is important for patients with aniridia.  Early institution of physical therapies for patients with developmental delay should be able to improve their long-term prognosis.
